# Supplementary material for: Conserved transcriptomic profile between mouse and human colitis allows unsupervised patient stratification
Source: Nat Commun. 2019 Jun 28;10:2892. doi: 10.1038/s41467-019-10769-x (PMC6598981; doi:10.1038/s41467-019-10769-x)
Supplement: Supplementary file 1 — Supplementary information [file 41467_2019_10769_MOESM1_ESM.pdf]

Conserved transcriptomic profile between mouse and human colitis allows unsupervised patient stratification

Czarnewski et al.

Figure S1

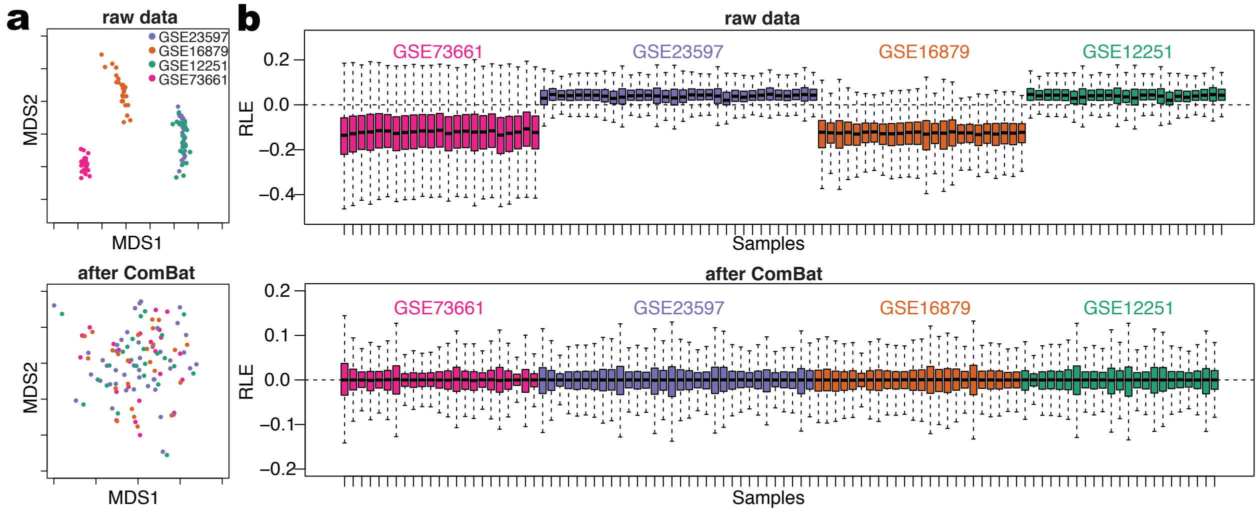

Figure S1. Normalization of publicly available ulcerative colitis datasets. (**a**) Multidimensional scaling (MDS) plots before and after batch effect correction using ComBat. x- and y-axis represent the first and second dimentions (MDS1 and MDS2). (**b**) Relative log expression (RLE) plots comparing samples from the different datasets before and after adjusting for batches using ComBat. Each sample is individually represented as a boxplot represented as the median (center line, 2nd quantile), 1st and 3rd quantiles (box) and whiskers extending 1.5 times interquartile range (IQR).

Figure S2

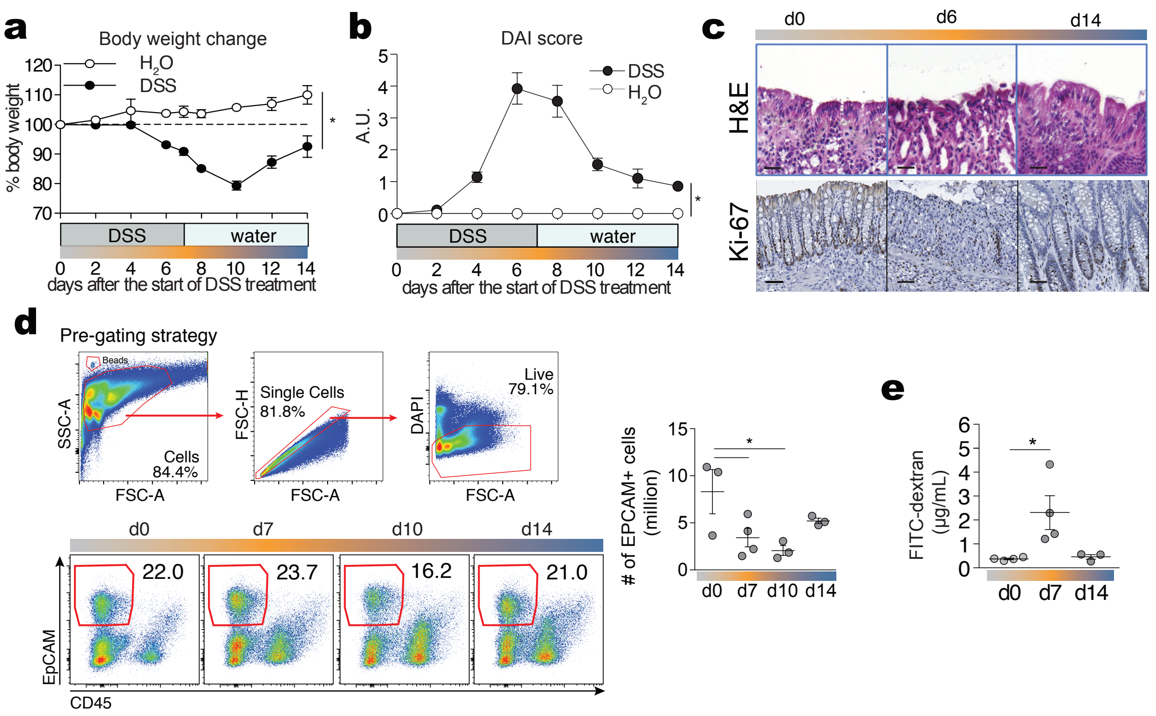

Figure S2. Macroscopic alterations in mice during DSS-induced colitis. (**a**) Body weight change over the time course of colitis. *P < 0.05; two-way ANOVA. (**b**) Disease activity index score (DAI) over time (in arbitrary units, A.U.). *P < 0.05; two-way ANOVA. (**c**) Representative histological section of the colonic tissue at indicated time points. H&E (upper) and immunohistochemistry staining for Ki-67 (bottom) are depicted. One representative figure out of three experiments. Scale bar 50 µm. (**d**) Flow cytometry data showing colonic epithelial cell (EpCAM+CD45-) frequencies during the course of the experiment. Cells were pre-gated on singlets and DAPI negative population. Dot plots are representative of three experiments. The graph on the right shows epithelial cell absolute numbers during the course of the experiment. *p < 0.05; two-way ANOVA. (**e**) Quantification of intestinal permeability by FITC-dextran assay. Mice were gavaged with 10 mg/mL of FITC-dextran and sacrificed 4 hours later for quantification of fluorescence in the serum. *p < 0.05; two-way ANOVA. Error bars represent SEM.

Figure S3

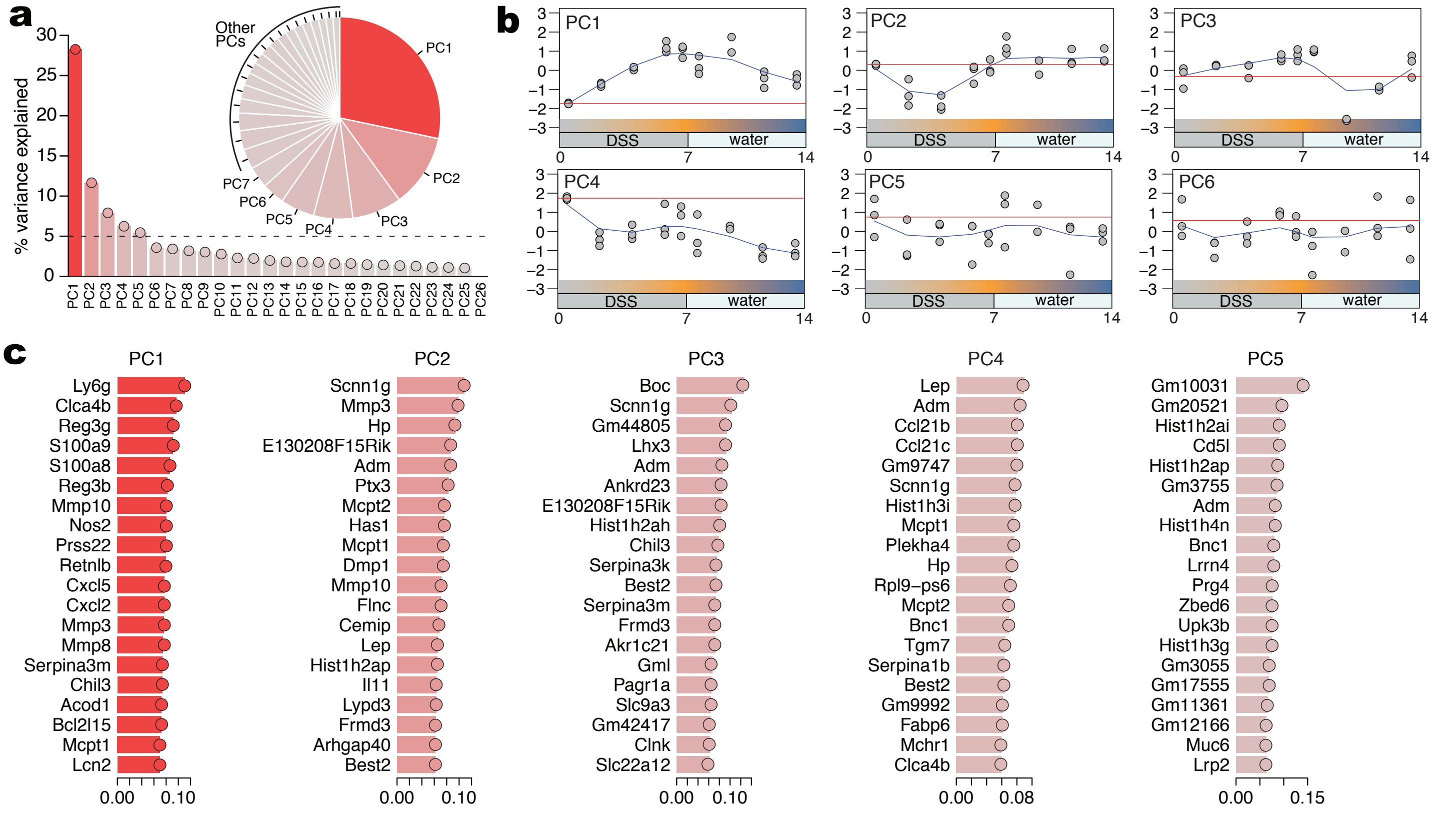

Figure S3. Identification of top leading genes and that drive overall differences in gene expression during DSS colitis. (**a**) Percentage of variance explained by each principal component (see Fig 2b). (**b**) Overall fluctuations in the first 6 PCs over the time course of DSS colitis. Note that the overall variance captured by PC6 is close to 0 and therefore not used in further analysis. (**c**) Ranking of the top 20 leading genes that contribute to the variance in each of the first 5 PCs.

Figure S4

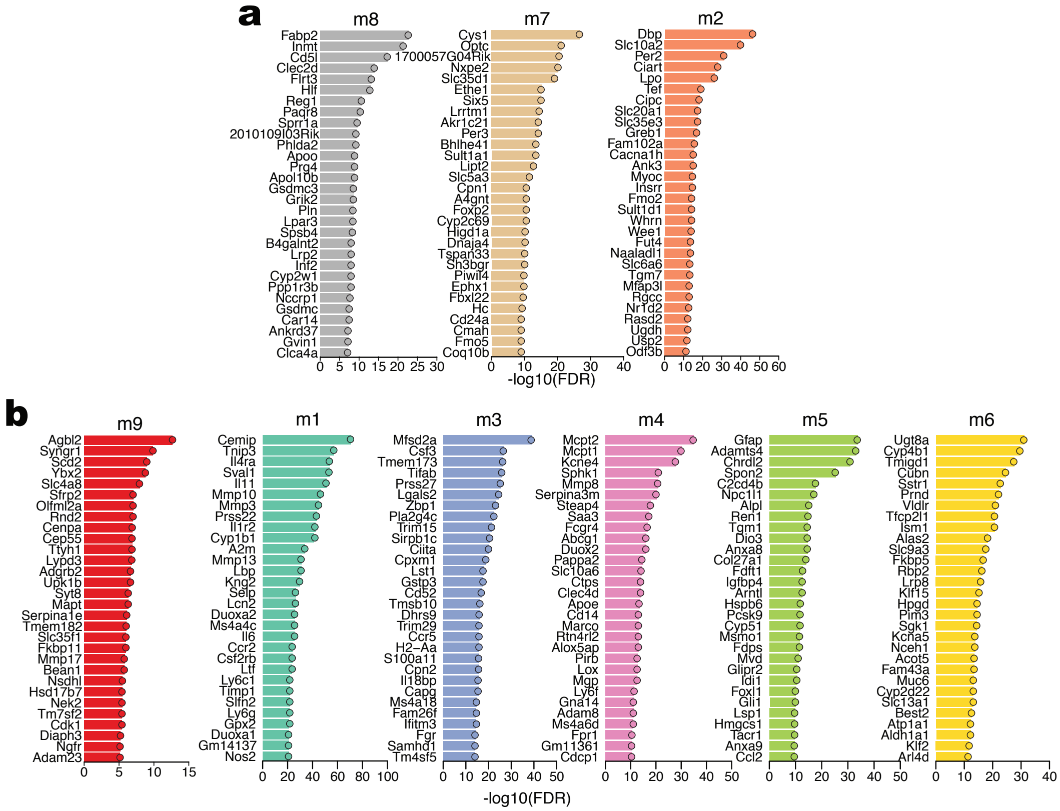

Figure S4. List of the top DEGs per module. (**a**) Down-regulated gene modules m8, m7 and m2 (see Fig 2c). (**b**) Up-regulated gene modules m9, m1, m3, m4, m5 and m6 (see Fig 2c).

Figure S5

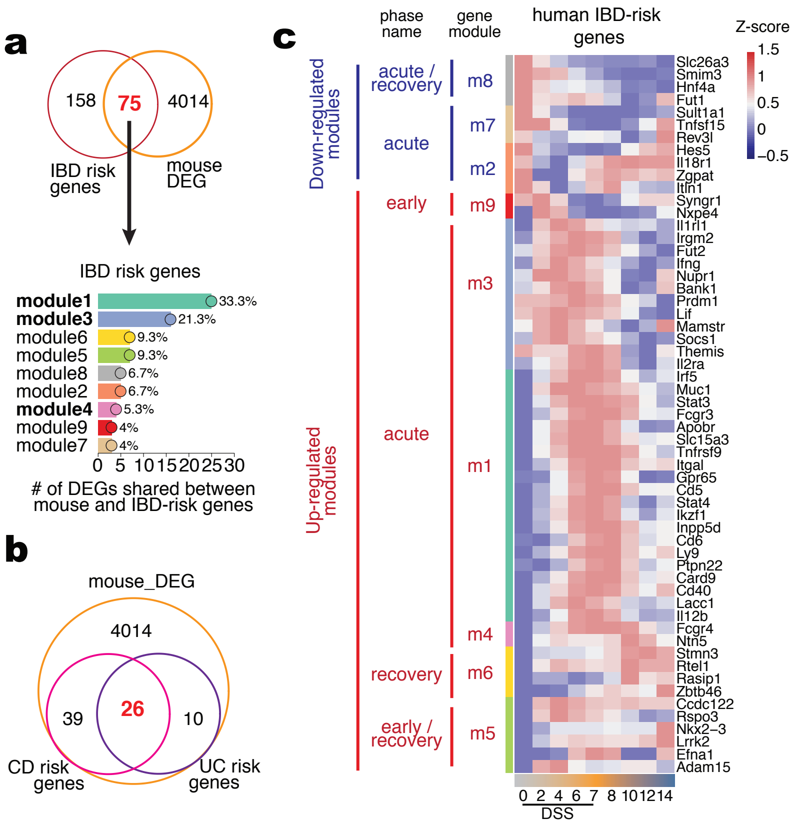

Figure S5. Mouse colitis and human UC share inflammatory pathways and IBD risk genes. (**a**) Venn diagram comparing IBD risk genes and the list of DEGs in the mouse dataset (upper). 75 genes are shared between these lists (in red). The number and percentage out of the 75 IBD-risk genes presented in each mouse module is shown (below). Modules highlighted in bold are the ones enriched for inflammatory terms in Fig 2c. (**b**) Venn diagram for the genes in the list of DEGs in the mouse dataset, genes associated with UC and/or to CD. Among those, 26 are shared between UC and CD (in red). (**c**) Expression level of IBD risk gene mouse homologs during the DSS colitis.

Figure S6

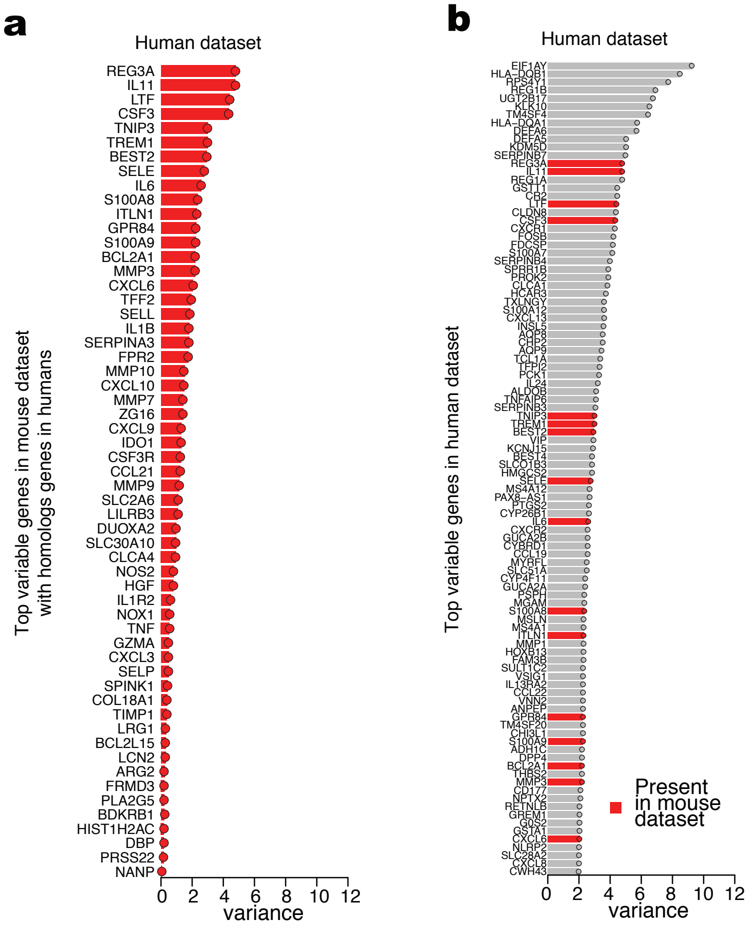

Figure S6. List of highly variable genes in humans and mouse colitis. (**a**) Top list of homolog genes identified in the mouse colitis dataset, sorted by variance on the human dataset. (**b**) Top 100 genes sorted by high variance in the human dataset. Genes highlighted in red are also present among the top list of homolog genes identified in the mouse colitis dataset.

Figure S7

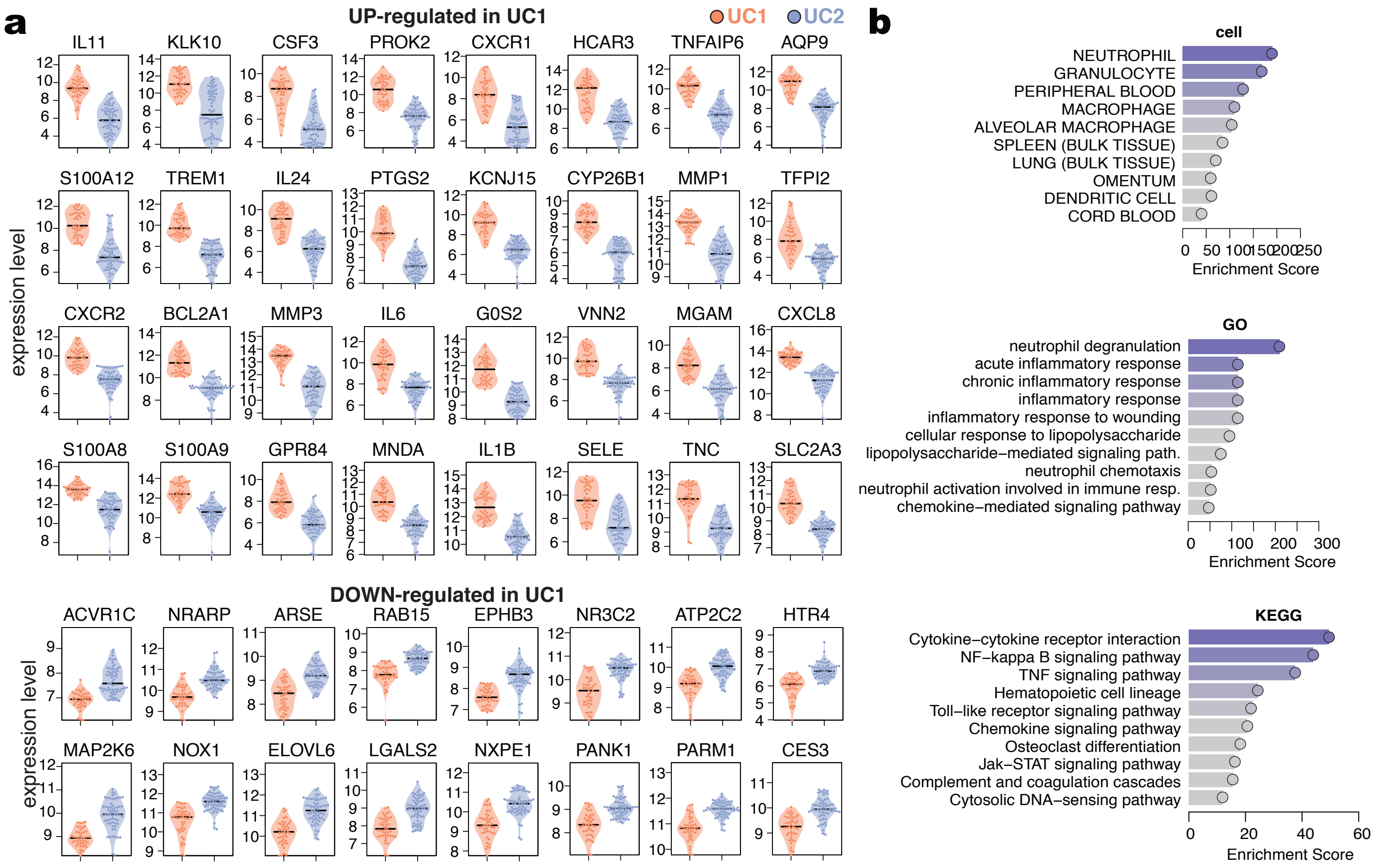

Figure S7. List of DEGs between UC1 and UC2 and enrichment analysis. (**a**) List of the top 32 up-regulated and 16 down-regulated DEGs between UC1 and UC2. (**b**) Cell, GO and KEGG enrichment analysis for the genes up-regulated in UC1 compared to UC2.

Figure S8

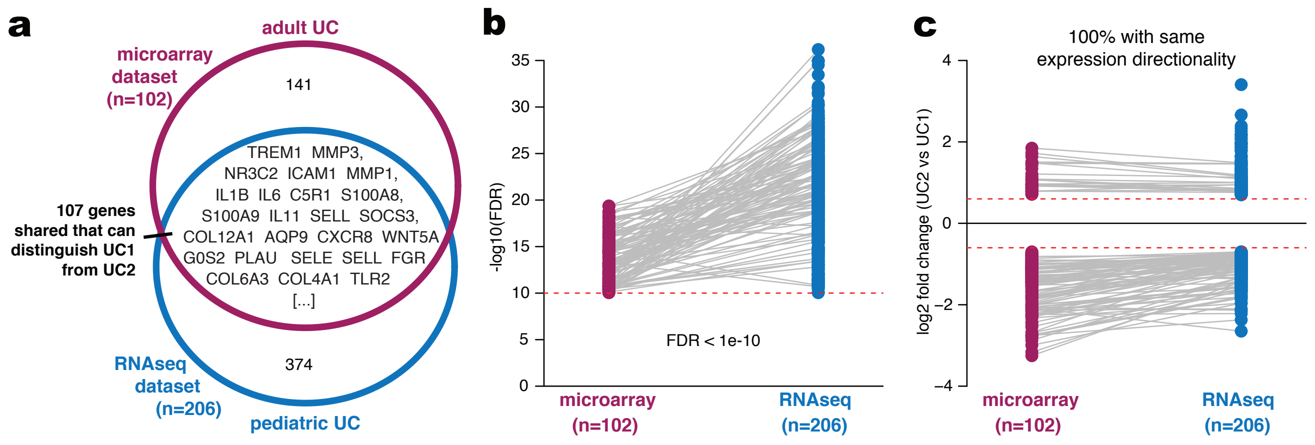

Figure S8. Comparison of DEGs between microarray (adult UC) and RNA-seq (pediatric UC) datasets for identification of UC1 and UC2. (**a**) Venn diagram showing the list of DEGs found between UC1 and UC2 identified using the microarray (n=102) or the RNA-seq (n=206) dataset. The same stringent cut-off was used for definition of differentially expressed genes in both datasets (FDR < 1e-10 and log2(FC) > 1). 107 genes were shared between analysis and some of the genes found are shown for illustration. (**b**) Comparison of p-values for the DGEs found in both datasets, showing where the top DEGs identified in the microarray dataset lie in the RNAseq dataset. Grey lines connect the same gene in each dataset. (**c**) Comparison of log fold changes in expression of DEGs found between datasets, showing that 100% of the DEGs found preserve their expression directionality. Grey lines connect the same gene in each dataset.

Figure S9

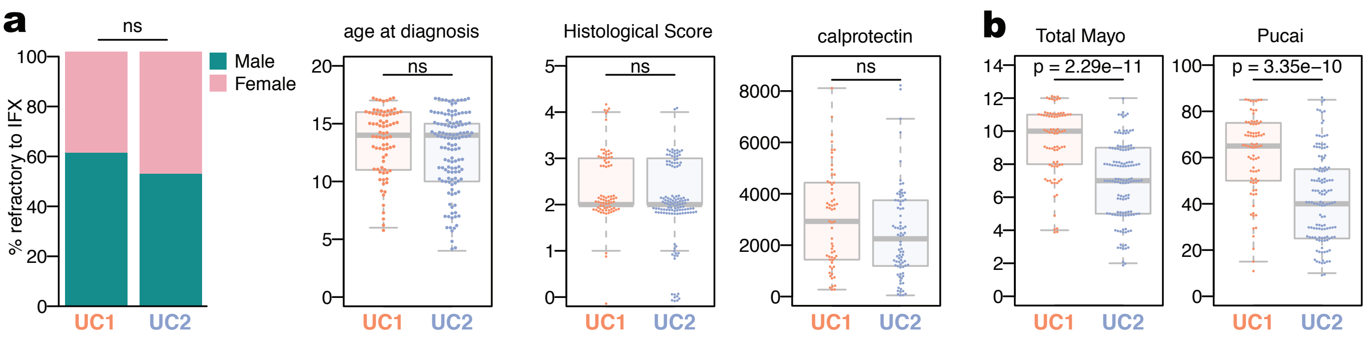

Figure S9. UC1 and UC2 profiles are associated with total Mayo and Pucai scorings, but not age, histological score or calprotectin levels. (**a**) Sex distribution, age at diagnostics, histological score and calprotecting level comparison between UC1 and UC2. (**b**) Total Mayo and Pucai scoring for UC1 and UC2 patients. Samples were compared with non-parametric Mann-Whitney test or Chi-square test (for sex distribution). To avoid overcrowding, a small random number was added to each value to allow visualization of each individual patient point in the plots, but not in the statistical tests. Each sample is individually represented as a boxplot represented as the median (center line, 2nd quantile), 1st and 3rd quantiles (box) and whiskers extending 1.5 times interquartile range (IQR).
