# Supplementary material for: Conserved transcriptomic profile between mouse and human colitis allows unsupervised patient stratification
Source: Nat Commun. 2019 Jun 28;10:2892. doi: 10.1038/s41467-019-10769-x (PMC6598981; doi:10.1038/s41467-019-10769-x)
Supplement: Supplementary file 12 — Reporting Summary [file 41467_2019_10769_MOESM12_ESM.pdf]

nature research

Corresponding author(s): DBPR (NCOMMS-19-00868A)

Last updated by author(s): May 16, 2019

Reporting Summary

Nature Research wishes to improve the reproducibility of the work that we publish. This form provides structure for consistency and transparency in reporting. For further information on Nature Research policies, see Authors & Referees and the Editorial Policy Checklist.

Statistics

For all statistical analyses, confirm that the following items are present in the figure legend, table legend, main text, or Methods section.

n/a	Confirmed	
☐	☒	The exact sample size (*n*) for each experimental group/condition, given as a discrete number and unit of measurement
☐	☒	A statement on whether measurements were taken from distinct samples or whether the same sample was measured repeatedly
☐	☒	The statistical test(s) used AND whether they are one- or two-sided<br>*Only common tests should be described solely by name; describe more complex techniques in the Methods section.*
☒	☐	A description of all covariates tested
☐	☒	A description of any assumptions or corrections, such as tests of normality and adjustment for multiple comparisons
☐	☒	A full description of the statistical parameters including central tendency (e.g. means) or other basic estimates (e.g. regression coefficient) AND variation (e.g. standard deviation) or associated estimates of uncertainty (e.g. confidence intervals)
☒	☐	For null hypothesis testing, the test statistic (e.g. *F*, *t*, *r*) with confidence intervals, effect sizes, degrees of freedom and *P* value noted<br>*Give P values as exact values whenever suitable.*
☒	☐	For Bayesian analysis, information on the choice of priors and Markov chain Monte Carlo settings
☒	☐	For hierarchical and complex designs, identification of the appropriate level for tests and full reporting of outcomes
☒	☐	Estimates of effect sizes (e.g. Cohen's *d*, Pearson's *r*), indicating how they were calculated

Our web collection on statistics for biologists contains articles on many of the points above.

Software and code

Policy information about availability of computer code

Data collection

Mouse sequencing data was generated in our laboratory.
Human microarray and RNA-seq gene expression matrices were obtained from Gene Expression Omnibus using the GEOquery package in R (freely available).

Data analysis

All analysis were done using open source software and R programming language using custom code and free packages.

Mouse gene expression by mRNA sequencing
Samples were sequenced using single-end 50bp sequencing, aiming an coverage of 20M reads. Read quality was inspected using MultiQC45, trimmed with Trimmomatic46 and further proceeded for abundance estimation using Kallisto.
Further data analysis was done in R programming language (Rstudio). Genes with absolute read count less than 5 in at least 3 samples were considered with low expression and filtered out. Differences in tissue cell composition that could affect transcriptional pools were balanced by means of removing unwanted variation based on negative control genes using the RUVg function implemented in RUVseq package48. Analysis revealed that library sizes strongly correlated with several known intestinal housekeeping genes, such as Hprt (r=0.87) and Gapdh (r=0.85), but not Actb (r=0.68). Moreover, genes such as Cd63 (0.94), Trappc (r=0.97), and Cpped1 (0.97) and Slc25a3 (r=0.96) correlated even more strongly to the library sizes, indicating potentially novel housekeeping genes during colonic inflammation. Negative controls genes were thus defined as genes with positive Pearson correlation above 0.9 to their respective sample library sizes. Estimated unwanted variation vectors were then used as covariates for calculation of differentially expressed genes (DEGs) using EdgeR package. EdgeR is specialized in performing time-series differential expression by means of generalized linear model (glm) function, where time points were parsed as independent factors in the contrast matrix, thus allowing detection of differentially expressed genes at any given time point. Genes were considered differentially expressed when the overall false discovery rate (FDR) < 0.01 and at least one time-point had fold change > 1.5. DEGs identified in this manner were used for dimensionality reduction by principal component analysis (PCA), from which gene-wise contribution to the total variation can be calculated.
Identification of gene modules was done based on smoothed temporal expression curves50. Briefly, gene-wise log fold changes were smoothed using spline curves and further grouped into modules by using Pearson correlation as distance for hierarchical agglomerative clustering with Ward's method ("ward.D2"). Functional gene annotation was performed on each gene module individually using the

Gene Ontology (GO_Biological_Process_2017) and the Kyoto Encyclopedia of Genes and Genomes (KEGG_2016) libraries with enrichR package51.

UC and IBD risk gene mapping to the murine RNA-seq dataset
To identify which genes are shared between mouse and human ulcerative colitis, we compared the list of DEGs identified by in the DSS dataset and the list of genes identified by Taman et al.26. Mapping of IBD risk genes was done using the list of IBD risk genes identified by fine-mapping at the single loci resolution 10. Identification of enriched GO processes and KEGG pathways was done using enrichR51.

Classification of adult UC molecular subtypes
To investigate whether the nuances of inflammation observed in the mouse model could also be found in humans, we made use of four human microarray datasets from GSE1225113, GSE7366115, GSE2359712 and GSE1687914. Combined, these datasets contain gene expression and metadata of 447 patients, containing information such as disease type (UC or CD), Mayo macroscopic score, the therapy given, when the sample was collected and the response to infliximab (IFX) or to vedolizumab (VDZ). Across all datasets, patients were considered inflamed if presenting a Mayo score of 2 or 3 (out of 3). Similarly, patient were considered to respond to therapy when it respective Mayo score reduced to 0 or 1, between 4-8 weeks of treatment with IFX or between 6-52 weeks of treatment with VDZ. For this study, we included only patients with UC before receiving any therapy (either IFX or VDZ), comprising a total transcriptional profiles of 143 patients, of which 102 received IFX and 41 for VDZ.
Probes with log2 fluorescence count lower than 6 in at least 10 samples were excluded from the analysis. Batches between dataset were observed and corrected using the ComBat function in SVA package52. Selection of genes for further exploration was done by different approaches: 1) using all genes; 2) using only the top 100 highly variable genes; 3) using the genes with top 100 high dispersion; 3) The gene with high loading in principal component 1 and; 4) The gene with high loading in principal component 2.
We determined whether clustering patterns exist by 4 independent methods: 1) By dimensionality reduction using tSNE. Since data originated from biopsies are known to present high variability across patients22, dimensionality reduction and visualization was done using t-Stochastic neighbor embedding (t-SNE). Because of it's nonlinear characteristics, t-SNE becomes less sensitive to noise and outperform PCA53 to discriminate biopsies based on shared expression patterns, rather than their absolute expression values.; 2) By visual assessment of clustering tendency (VAT) using dissimilarity matrices16; 3) By using the Hartigan's dip test19,20, which tests whether the gene distribution are different to an unimodal distribution. Values close to 1 indicate that the data is unlikely to present cluster substructures. We performed bootstrapping 100 times on 90% of the samples to calculate Hartigan's dip test p-value. The comparison between bootstrapping with human highly variable genes and mouse PCs (see below) was done using paired Mann-Whitney test; 4) By dividing patients into subgroups using hierarchical agglomerative clustering. Cluster stability was determined by bootstrapping 300 times on 90% or the samples, resulting in the approximate unbiased (AU) statistics21. Clusters with AU closer to 100 present higher stability.
Instead of using the top variable genes as above, we alternatively used the top genes identified in the mouse RNA-seq DSS colitis dataset (see above). To this end, the top 100 genes identified in PC1 and PC2 were selected for identification of the respective human homologs. Together, 175 genes were found in top genes in both PC1 and PC2 and from these, 148 genes had a homolog in humans. In total, 57 homolog genes were found between our mouse PCs and the human dataset. Dimensionality reduction was performed with tSNE. Assessment of clustering tendency was done as described above. Agglomerative clustering on the Euclidean distance using complete linkage was used to discriminate patient subgroups UC1 and UC2. For the matter of definition used in this study, patients that present higher mean expression of the 57 mouse-human homologs were classified as UC1, while those with low expression were classified as UC2. Differences in expression between UC1 and UC2 were calculated using eBayes method in limma package54. Probes with fold changes above 1.5 and FDR lower than 0.001 were considered significantly differentially expressed. Identification of enriched GO, KEGG and cell types was done using enrichR51.
To identify which genes can discern UC1 from UC2, we trained a logistic regression classifier for each gene individually and comparing to the UC1 and UC2 classification mentioned above. The sensibility and sensitivity of the prediction was summarized using the area under the curve (AUC) method. Genes with AUC values closer to 1 (100%) have a better accuracy to distinguish UC1 and UC2 patients.

Classification of UC molecular subtypes in peditric patients
In addition to using mouse genes to stratify adult UC patients (see above), we applied a similar strategy to a RNA-seq dataset from pediatric UC patients 33. This dataset contains the expression levels and detailed metadata information of 206 colonic samples. After failing to respond to first line therapy, all patients in this cohort received infliximab and the response was evaluated after 4 weeks. Genes with read count less than 5 in at least 10% of the samples were considered with low expression and filtered out. Batches associated with sex chromossomes were detected and corrected using ComBat 52. Counts were normalized by TMM normalization method implemented in EdgeR package 49, and subsequently used for stratification using the genes in PC1 and PC2 identified in mouse model of colitis (see above). EdgeR and limma packages estimate differential expression by different assumptions, and therefore result in slitly different results 55. Thus, to allow fair comparison between the results found between the microarray dataset and the RNA-seq, we opted to use the same differential expression strategy for in both datasets. Differences in expression between UC1 and UC2 were calculated using eBayes method in limma package54 using log2 transformed counts per million (instead of raw counts). Strict cutoffs were used to ensure result robustness that also accounts for the differences in sample size and methodologies between the datasets. Genes with fold changes above 1.5 and FDR lower than 1-10 were considered significantly differentially expressed. Comparisson between both dataset were done using Venn diagrams, and by comparing FDR statistics and log2 fold changes in gene expression. Finally, differences in metadata parameters between UC1 and UC2 were evaluated using Chi-square or with Mann-Whitney tests when applicable.

All code used is available from the authors.
Codes used are available on Github (https://github.com/czarnewski/uc_classification).

For manuscripts utilizing custom algorithms or software that are central to the research but not yet described in published literature, software must be made available to editors/reviewers. We strongly encourage code deposition in a community repository (e.g. GitHub). See the Nature Research guidelines for submitting code & software for further information.

Data

Policy information about availability of data

All manuscripts must include a data availability statement. This statement should provide the following information, where applicable:

- Accession codes, unique identifiers, or web links for publicly available datasets
- A list of figures that have associated raw data
- A description of any restrictions on data availability

All mouse sequencing data used in the manuscript was deposited at the Gene Expression Omnibus (GEO) under accession code GSE131032. All other relevant data is available from the authors.

All human gene expression data used was obtained from GEO (GSE1225113, GSE7366115, GSE2359712, GSE1687914 and GSE109142).

Field-specific reporting

Please select the one below that is the best fit for your research. If you are not sure, read the appropriate sections before making your selection.

☒ Life sciences ☐ Behavioural & social sciences ☐ Ecological, evolutionary & environmental sciences

For a reference copy of the document with all sections, see nature.com/documents/nr-reporting-summary-flat.pdf

Life sciences study design

All studies must disclose on these points even when the disclosure is negative.

Sample size	Sample sizes for mouse colon RNA-seq were estimated according to [1]. Due to robustness of RNA-seq methodology, 3 samples per time point were used. Differential gene expression was done using EdgeR, which performed best with such sample numbers [1].<br>1. Ching T, Huang S and Garmire LX. Power analysis and sample size estimation for RNA-Seq differential expression. RNA. 2014 Nov; 20(11): 1684–1696. doi: 10.1261/rna.046011.114<br><br>For all other mouse experiments (e.g. FACS, Histology and FITC-dextran assay), 3-4 mice were used per group per experiment. Each experiment has been repeated 2-3 independent times to ensure result reproducibility.<br><br>For human dataset analysis (from public deposited data), all samples were used.
Data exclusions	No data sample was excluded. Only in the case of failed due to technical problems, e.g. failure to isolate intestinal cells.<br>No data sample was excluded from human dataset analysis (from public deposited data).
Replication	All attempts of replication of mouse experiments were successful.
Randomization	Mice were randomly assigned to to groups and cages, in order to balance cage effects.
Blinding	Histological scoring was performed blinded.

Reporting for specific materials, systems and methods

We require information from authors about some types of materials, experimental systems and methods used in many studies. Here, indicate whether each material, system or method listed is relevant to your study. If you are not sure if a list item applies to your research, read the appropriate section before selecting a response.

Materials & experimental systems

n/a	Involved in the study	
☐	☒	Antibodies
☒	☐	Eukaryotic cell lines
☒	☐	Palaeontology
☐	☒	Animals and other organisms
☒	☐	Human research participants
☒	☐	Clinical data

Methods

n/a	Involved in the study	
☒	☐	ChIP-seq
☐	☒	Flow cytometry
☒	☐	MRI-based neuroimaging

Antibodies

Antibodies used	CD45.2-PE.Cy7 (BioLegend, Cat# 109829, clone 104)<br>EPCAM-FITC (BioLegend, Cat# 118202, clone G8.8)

	biotinylated goat anti-rabbit secondary antibody (Vector Labs, Cat# BA-1000, polyclonal)<br>Ki67 Monoclonal Antibody (Thermo Fisher, Cat# MA5-14520, clone SP6)
Validation	All antibodies are quality tested by manufacturers.<br>Below is the links to the page of used antibodies, containing the validation reports:<br><br>https://www.biolegend.com/en-us/products/pe-cy7-anti-mouse-cd45-2-antibody-4918<br>https://www.biolegend.com/en-us/search-results/purified-anti-mouse-cd326-ep-cam-antibody-4724<br>https://vectorlabs.com/biotinylated-goat-anti-rabbit-igg-antibody.html<br>https://www.thermofisher.com/antibody/product/Ki-67-Antibody-clone-SP6-Monoclonal/MA5-14520

Animals and other organisms

Policy information about studies involving animals; ARRIVE guidelines recommended for reporting animal research

Laboratory animals	Eight to twelve weeks old C57BL/6J female mice were used. Mice were obtained from ScanBur.
Wild animals	The study did not involve wild animals.
Field-collected samples	The study did not involve Field-collected samples.
Ethics oversight	Animal experiments were done following institutional guidelines of the Stockholm Regional Ethics Committee under approved ethical permit number N89/15.

Note that full information on the approval of the study protocol must also be provided in the manuscript.

Flow Cytometry

Plots

Confirm that:

☒ The axis labels state the marker and fluorochrome used (e.g. CD4-FITC).

☒ The axis scales are clearly visible. Include numbers along axes only for bottom left plot of group (a 'group' is an analysis of identical markers).

☒ All plots are contour plots with outliers or pseudocolor plots.

☒ A numerical value for number of cells or percentage (with statistics) is provided.

Methodology

Sample preparation	Cell isolation from the colonic tissue was performed as previously described 56 with modifications. Briefly, tissues were open longitudinally, cut into 1cm pieces and washed with PBS. The epithelial cell fraction was obtained by incubating the tissue with Buffer-A (PBS, 5% FCS, 5 mM EDTA) at 37℃ for 20 minutes under agitation at 600 rpm. The supernatant was collected and kept on ice while the remaining tissue was washed 2 times with PBS. Tissue were digested with collagenase solution containing 0.15 mg/ml Liberase TL (Roche) and 0.1mg/ml DNase I (Roche) in HBSS and incubated at 37℃ for 60 minutes under agitation at 1200 rpm. The digested and the epithelial cell fraction were mixed, filtered through a 100 um cell strainer, pelleted by centrifugation at 1750 rpm and re-suspended in Buffer-A. Cell suspensions were blocked with Fc-blocking solution (1:1000, eBioscience) and stained with the antibody mix (1:200), both at 4℃ for 15 minutes. The following antibodies were purchased from BD Biosciences: CD45.2 (104) and EPCAM (G8.8). Counting beads (Spherotech) and DAPI (1:400, Sigma) were added to each sample to allow absolute cell quantification and exclusion of dead cells.
Instrument	Samples were acquired on an LSR Fortessa flow cytometer (BD Bioscience)
Software	Data acquisition was done using 5-laser LSR Fortessa flow cytometer with FACS Diva software (BD Biosciences) and analysis was carried out with FlowJo software (TreeStar).
Cell population abundance	No sorting was performed in this study.
Gating strategy	Gating on FSC-A / SSC-A was done to select cell-sized events, which were further submitted for FSC-A / FSC-H gating for selection of single cells. Next, events were applied to DAPI / FSC-A gating for exclusion of dead cells. After this step, cells were then gated on EPCAM / CD45 gating, where EPCAM+DAPI- cells are epithelial cells, CD45+EPCAM- cell are immune cells and CD45-EPCAM- are stromal cells.

☒ Tick this box to confirm that a figure exemplifying the gating strategy is provided in the Supplementary Information.
